# Abstracts of the 19th International Conference on Radionuclide Therapy (ICRT), World Association of Nuclear Medicine, Muscat, Oman, 8–12 February, 2024

**DOI:** 10.1055/s-0044-1782143

**Published:** 2024-04-08

The World Association of Radiopharmaceutical Therapy (WARMTH) held a 5-day event, International Congress on Radiopharmaceutical Therapy (19 ^th^ ICRT), 8 to 12 February 2024 in Muscat, Oman. There were more than 80 presentations in the Congress program. Most of these published abstracts were presented as posters.

The objectives of the conference were to evaluate the current status of radiopharmaceutical therapy in the world in general, to exchange information on the current advances in the field between scientists from developed and developing countries, to interact with user groups (clinicians, oncologists, surgeons, radiopharmacists, medical physicists, etc.) and to bring them the most important information in the field, and to define future directions for the speciality.

The conference covered several topics and discussed many issues covering all aspects of radiopharmaceutical therapy including several plenary lectures. The titles of the sessions included 1) Ajit Padhy Oration, 2) New Trends in Oncology and Precision Medicine, 3) Pediatric Nuclear Medicine, MIBG imaging, 4) Thyroid Imaging and Treatment: What is new?, 5) Prostate Cancer (PRLT) - newest developments, 6) Peptide Receptor Therapies, 7) Radioembolization and Liver Therapies, 8) Locoregional treatments, 8) Nuclear Cardiology, 9) Bone Targeted Therapies, 10) Radiosynovectomy, and 11) miscellaneous topics.

World J Nuclear Med 2024;23:57–70.

## Theragnostics

#### A001. Evaluation of Production Data of Neodymium-140; A therapeutic Radionuclide

H. Rasheed[1]
[1]University of Education, Lahore, Pakistan

During a search for new nuclides that can be used for treatment, it was discovered that $^{140}$Nd (T1/2 = 3.4d, 100% EC, no radiation) has unique properties that make it suitable for therapy. The only electrons emitted by the nuclide are Auger electrons. Its half-life is suitable for endo radiotherapy as well. Its daughter nucleus $^{140}$Pr (T1/2 = 3.4 minutes, E+ = 2.4 MeV) is extremely useful for positron emission tomography studies in vivo. In this study, the evaluation of therapeutic radionuclide $^{140}$Nd has been done. We adopted a well-defined evaluation technique where experimental data are compiled and calculated cross-section data by using theoretical model codes, that is, EMPIRE 3.2, ALICE-IPPE and TALYS 1.9 to generate a recommended fit. Using this recommended fit thick target yield of desired reaction is measured. For the impurities analysis thick target yield of selected radionuclides compared with corresponding impurities. Then energy range drawn for the optimum production of selected radionuclide. Comparison of radionuclides production by using different projectile can be also done for suggesting of best possible production route.

#### A002. Radiosynoviorthesis with Tin-117m Colloid in a Canine Model for Osteoarthritis

P. Cohen[1], N. Stevenson[2]
[1]Lion's Gate Hospital, Canada
[2]Exubrion LLC, United States

Tin-117m is a novel theranostic compound. It not only emits a 159 Kev photon compatible with existing single-photon emission tomography gamma cameras for imaging, but also has a short-range (0.3 mm) monoenergetic 127 to 158 Kev for therapy, similar to α particles. It has a 14-day half-life. It is proposed to use it for radiosynoviorthesis for rheumatoid and osteoarthritis in humans, but has been approved in the past 2 years for the treatment of canine osteoarthritis in the United States. We report on the benefits of this therapy in canines and the potential for the treatment of human osteoarthritis.

#### A003. Design of Antidisialoganglioside GD2 Antibody as a Nuclear Imaging Probe for Neuroblastoma Xenografts: A Theranostic Proof of Concept

C. Yang[1], Y. Khor[1], A. Loh[2]
[1]Singapore General Hospital, Singapore
[2]KK Women's and Children's Hospital, Singapore

*Introduction:* High-risk childhood neuroblastoma patients have poor survival outcomes with few treatment options for relapse or refractory disease. A new treatment approach is targeted immunotherapy using a monoclonal antibody against a tumor-associated antigen, the disialoganglioside GD2, but the treatment has side effects and is costly. Development of a noninvasive tool to image and quantity the presence of the GD2 tumor antigen may help in patient selection. The aim of this study is to label anti-GD2 antibody with radioiodine and analyze the biodistribution in neuroblastoma–bearing mice.

*Methods:* All experiments were done in compliance with, and with the approval of, the institutional animal care and use committee. The monoclonal chimeric antibody targeting the GD2 antigen, APN311, was conjugated to radioactive iodine using the chloramine-T approach with radiolabeling yield 73 to 92% [1]. The radio-iodinated APN311 was then purified with PD 10 column to achieve high purity (>96%). The stability testing using thin layer chromatography showed the 131I-APN311 is stable in PBS up to 3 days. Biodistribution studies were performed 6, 24, 48, and 72 hours after injection of 15 to 20 MBq 131I-APN311 into BALB/c mice bearing high and low GD2-expressing patient derived neuroblastoma xenografts.

*Results:* APN311 was radiolabeled with high efficiency and isolated in high purity. Biodistribution studies showed excellent radioactivity targeting high GD2 expressing tumor with high tumor-to-normal tissue ratio (tumor to blood ratio at 48 hours was 3.9:1 and at 72 hours was 9.1:1). Uptake in high GD2 expressing tumor was more than threefold higher than in low GD2 expressing tumor at 48 hours (11.4 vs. 3.2% ID/g) and 72 hours (5.6 vs. 1.2%ID/g).

*Conclusion:* In our preclinical model, 131I-APN311 displays high tumor-to-normal tissue contrast for the detection of GD2 tumor antigen in patient-derived neuroblastoma xenografts. Our study strongly suggests the clinical utility of radio-iodine-labeled APN311 in assessing GD2 expression status.

#### A004. A Comparison of Labeling Characteristics of Manual, Lyophilized-Kit, and Automated Synthesis Methods for 177Lu-PSMA

S. Sibiya[1], S. du Plessis[1], J. le Roux[2], S. Rubouw[2]
[1]NTP Radioisotopes SOC Ltd, South Africa
[2]University of Stellenbosch, South Africa

*Introduction:* The devastating reach of prostate cancer (PC) can be noted worldwide. PC is rated as the second most common cancer occurring in males and the most prevalent cancer affecting men in South Africa according to CANSA SA. Global data from 2018 indicates that as many as 1.2 million per year were diagnosed with PC with over 35,000 deaths (Sadaghiani et al 2021). It is further noted that the disease is the most common cancer for males aged between 60 and 79 years. Due to the nature of the disease progression, treatment of PC is challenging and as such new therapeutic modalities to be employed for patients with PCs are sought (Sadaghiani et al 2021). Since its introduction, labor-intensive methods have been heavily relied on to synthesize lutetium-177 using peptides such as DOTA-TATE and now PSMA. A drawback of using a manual method is requiring a skilled operator to do the manual manipulations. This of course comes with the added effect of high radiation burden on the operator manually performing the manipulations that in turn can affect the ability to upscale a synthesis process that relies on this method of production. A manual method of synthesis is also notorious for the introduction of batch-to-batch variances. In line with current Good Manufacturing Principles (GMP) as well as Good Radiopharmaceutical Practice (GRPP), more systems are making a move from manual synthesis methods of lutetium-177 with peptides to more automated or lyophilized sterile-based synthesis methods. These alternative methods, when validated, can streamline processes and open possibilities of upscaling synthesis runs on a grand scale. These also solve several GMP nonconformances introduced by manual labeling by increasing process reliability and rigid conformance to the quality parameters set out for lutetium-177 peptide synthesis.

*Methods:* Synthesis of lutetium-177 PSMA utilizing a manual labeling method. Synthesis using the manual labeling method used a lutetium-177 n.c.a dose added to PSMA-I&T (purchased from ITM, Germany) and compounded using an ascorbic acid buffer to maintain a pH of 4.5 to 5.5. The reaction was incubated at 100°C for 25 minutes before the quality aspects outlined below were investigated for the product. Automated synthesis of lutetium-177 PSMA using ITG automated synthesis module A synthesis method for lutetium-177-PSMA-I&T labeling was validated for this study. An iQS automatic synthesis unit by iTM, Germany, was used to draw n.c.a lutetium-177-PSMA-I&T from the API vial having conditioned the C18 cartilage with ethanol and rinsed with water for injection. The n.c.a lutetium-177 was added to the reaction vial containing PSMA-I&T (ABX, Germany) and dissolved in a sodium ascorbate buffer before incubating the reaction at 100°C for 25 minutes. Synthesis of lutetium-177 PSMA utilizing a lyophilized sterile kit involves a lyophilized sterile kit of DOTA-iPSMA (ININ, Mexico), which comprises two kits, was used for this component of the study. Vial A (PSMA peptide) and Vial B (Buffer) were compounded with lutetium-177 n.c.a (NTP, South Africa) and incubated at 100°C for 25 minutes. The solution was then be filtered through a 0.22μm and dispensed in an end-product vial from which a quality control sample will be obtained and tested.

*Results:* The manual labeling method was able to yield a radiolabeled product that met specifications with a yield of 100%, an average pH of 4.5 and an RCP of 100%. Safety considerations for the method proved to be a challenge as contamination often exceeded 2mSv per labeling. The lyophilized kit method yielded an average of 98% radiolabeling yields batches achieving a baseline pH of 5. The average RCP for the batches averaged 100% with a stability profile exceeding 3 days. Radiological surveys for labeling averaged 1.5mSvi most of which resulted from the dispensing process. The automated synthesis unit showed more consistent labeling with minimal batch-to-batch variances, with an average radiolabeling yield of 95%, a pH of 5 an average RCP of 100%, with a stability profile exceeding 3 days. The contamination and exposure levels proved to be almost negligible with an average of less than 1mSvi per labeling.

*Conclusion:* Lutetium-177 radiolabeling with PSMA molecules such as PSMA-I&T as well as DOTA-iPSMA has proven to be robust and reliable when performed using a validated method. Labeling characteristics of the PSMA molecules evaluated have successfully met the required criteria for safety and quality as required of radiopharmaceuticals. The stability profile of the radiolabeled doses has shown that stability data previously reported for Lu-177 PSMA doses is stable for up to 3 days. Lyophilized kits when compared with manual labeling and automatic synthesis and manual labeling have shown to be cost-effective while providing the same level of quality and safety as the other two methods. Automated labeling of lutetium-177 doses has shown to be a more robust method, providing results that are consistent from batch to batch when compared with the other two methods. Manual labeling was able to provide for higher yields when compared with the other methods; however, the ability to derive more yields seemed to be dependent on the level of skill and experience of the operator. Each method has proven to be robust and reliable; the selection of an ideal method for any facility is dependent on the resources available in that facility, as guided by IAEA operational levels.

### Diagnostics

#### A005. A Retrospective Study on the Utility of Semi-Quantitative Analysis in Parathyroid Scintigraphy

**D. Alcantara[1], N. Hui[1], E. Lim[1]**
*[1]Nuclear Medicine and PET-CT Center, The Medical City, Philippines*

Lesion-to-background ratio (LBR) is the value of a lesion's radiation counts divided by the background counts, representing radiopharmaceutical uptake in nuclear medicine. Parathyroid LBR thresholds have not been established yet in the Philippines due to limited studies on preoperative parathyroid scintigraphy. To evaluate their utility in assessing the presence of hyperfunctioning parathyroids, the use of LBRs was correlated to surgical histopathologic assessment, parathyroid gland volume, ionized calcium, and intact parathyroid hormone (PTH). A retrospective analysis was done on 30 hyperparathyroidism patients in The Medical City who underwent technetium-99m sestamibi parathyroid scintigraphy with corresponding parathyroidectomy from January 2017 to June 2023. Histopathologic assessments were 25 parathyroid adenomas (83.3%), 2 parathyroid hyperplasia (6.7%), 1 normal parathyroid (3.3%), and 2 thyroid hyperplasia (6.7%). Early LBR of 3.7 and delayed LBR of 2.2 were the thresholds obtained with receiver operating characteristic curve analysis (area under the curve: 0.736 and 0.784), which respectively show a sensitivity of 63.0 and 77.8% and specificity of 66.7 and 66.7%. Moreover, Spearman analysis for early and delayed LBRs showed significant ($p < 0.05$) and moderate correlation with parathyroid gland volume (rho = 0.68 and 0.64), and fair correlation with intact PTH (rho = 0.45 and 0.48) and ionized calcium (rho = 0.50 for delayed LBR). The results of this single-center retrospective study demonstrate a direct relationship between LBR and presence of hyperfunctioning parathyroids. Use of LBR as an adjunct may improve reader confidence in the interpretation of the parathyroid scan.

#### A006. Diffuse Increased Kidney FDG Uptake in a Patient with Fever of Unknown Origin-Guided Biopsy and Assisted the Diagnosis of Microscopic Polyangiitis

**S. Vachatimanont[1], N. Bunnag[2], K. Phuangmali[3,4]**
*[1]Nuclear Medicine, King Chulalongkorn Memorial Hospital and International Program (CU-MEDi), Thailand*
*[2]Faculty of Medicine, Chulalongkorn University, Thailand*
*[3]Diagnostic Radiology Unit, King Chulalongkorn Memorial Hospital, Thailand*
*[4]Nuclear Medicine, Faculty of Medicine, Chulalongkorn University and Phramongkutklao College of Medicine, Thailand*

*Introduction:* Fluorodeoxyglucose F18 positron emission tomography/computed tomography (18F-FDG PET/CT) plays a pivotal role in investigating cases of fever of unknown origin (FUO), facilitating precise diagnoses and enabling effective treatments.

*Methods:* We present the case of an 80-year-old woman with FUO, where $^{18}$F-FDG PET/CT played a pivotal role in guiding kidney biopsy and subsequent diagnosis of microscopic polyangiitis (MPA).

*Results:* The patient presented with a 3-month fever without apparent causes. During the first month, she exhibited a transient rash, for which the skin biopsy showed nonspecific inflammatory changes without abnormal immunoglobulin deposits. Chest X-rays, echocardiography, magnetic resonance imaging of the entire spine, and CT angiography could not identify the cause of the fever. Blood tests revealed positive pANCA, anti-MPO, but negative cANCA and anti-PR3. Her serum creatinine rose from 0.59 to 4.17 mg/dL in 3 months, and the C-reactive protein (CRP) was elevated at 19.2 mg/L. $^{18}$F-FDG PET/CT was performed to identify the cause of the fever. In the 18F-FDG PET/CT, there was abnormally increased uptake in the kidney parenchyma, with a mean standardized uptake value (SUV) of 7.20 (mean liver SUV = 3.25). This finding is nonspecific and could be due to small vessel disease or infiltrative disease of the kidneys.

However, it led to a renal biopsy, which revealed pauci-immune crescentic glomerulonephritis with evidence of vasculitis. These fulfilled the classification criteria for MPA. Following the MPA diagnosis, the patient underwent treatment with corticosteroids and cyclophosphamide, leading to the resolution of fever in the subsequent month. Her CRP normalized to 4.66 mg/L 4 months after the treatment initiation and her serum creatinine stabilized at 2.4 to 2.6 mg/dL.

*Conclusion:* In this case, the $^{18}$F-FDG PET/CT could identify abnormalities that could not be detected by conventional imaging, leading to the kidney biopsy that was the crucial to establish the final diagnosis and appropriate treatment. Therefore, our report highlights the roles of 18F-FDG PET/CT in the evaluation of FUO.

#### A007. Tumor Recurrence versus Radiation Necrosis: $^{18}$F-FET and MRI Head-to-Head Comparison of Volumes on a Hybrid PET/MR System

**D. Sitani[1], D. Kumar[1], K. Sitani[1]**
*[1]National Institute of Mental Health and Neurosciences, Bengaluru, India*

*Introduction:* 18F-fluoro-ethyl-tyrosine (F18-FET), an amino acid radiotracer, is used in neuro-oncological imaging. Its use is more significant in those cases of high-grade gliomas where magnetic resonance imaging (MRI) is inconclusive in differentiating recurrence from treatment-related changes, since surgery, radiotherapy, and chemotherapy are the mainstays of treatment in this case. The study aims at observing and comparing the volumes of the lesions detected on three-dimensional (3D) fluid-attenuated inversion recovery (FLAIR), F18-FET PET, and gadolinium contrast-enhanced MRI (CE-MRI).

*Methods:* We analyzed a data of 46 glioma, post-treatment patients. All of them underwent F18-FET PET/MRI at our center. An activity of 5 to 7mCi (185–259 MBq) was injected. Scan was performed 20 to 30 minutes post-injection. MRI sequences taken were 3D FLAIR and T1-MPRAGE. PET imaging reconstruction was done using iterative reconstruction with 512 matrix, 5 iterations, FWHM 2. Volumes were calculated using the SYNGOVIA scenium version v.1, brain analysis software, where a VOI sphere was places in coronal, sagittal, and axial sections, on the visible recurrent lesion.

*Results:* Five out of forty-six cases showed no recurrence. In one of these cases, FLAIR changes were noted on the MRI with no FET uptake and contrast enhancement. In the rest of the 41/46 cases,1/46 showed no contrast enhancement since the beginning, showing FLAIR volume more than FET volume. In the rest of the 40/46 cases, 4/46 showed nearly similar volumes in all the three modalities. In 42/ 46 cases, FLAIR imaging showed the highest volumes. Comparing contrast enhanced with FET volumes in 40/46 cases, 7/40 cases showed FET volumes more than CE-MRI volumes and 33/40 cases showed it comparable to MRI. Results on comparison of diffusion and perfusion weighted imaging with FET are underway.

*Conclusion:* This study concluded that metabolic tumor volumes were more sensitive in detection of tumor volumes in the cases where no contrast enhancement was noted since the commencement or got resolved over time. Also, metabolic tumor volumes can be used for radiotherapy planning, considering they were more than contrast enhancing volumes in few cases.

#### A008. Presentation of Ovarian Metastases on F-18 FDG PET/CT

**R. Kumar[1], D. Datta[1]**
*[1]AIIMS, Jodhpur, Rajasthan, India*

*Introduction:* Ovarian metastases are infrequent ovarian neoplasms, and associated with poor prognosis. The route of spread can be hematogenous, lymphatic, transcoelomic, or direct in nature. Accurate diagnosis of the metastases and differentiation from primary ovarian malignancy is important in its treatment management and prognosis. We characterize the nature of radiologically identified ovarian metastases in terms of metabolic activity, laterality, and attenuation on fluorodeoxyglucose F18 positron emission tomography/computed tomography (F-18 FDG PET/CT).

*Methods:* This retrospective study was done from February 2021 to August 2022 at Nuclear Medicine Department, All India Institute of Medical Sciences (AIIMS), Jodhpur (India). Ethical clearance was obtained from Institutional Ethics Committee, AIIMS, Jodhpur. The patients with known nonovarian malignancies with increased FDG uptake in either or both ovaries were considered positive of ovarian metastases. The parameters recorded included laterality, attenuation (solid/cystic/mixed), and maximum standardized uptake value (SUVmax) of metastatic ovarian lesion. SUVmax was derived from the single image plane that had the highest SUV.

*Results:* Nine female patients met the inclusion criteria. Breast was the most common primary malignant site (78%). A total of 12 ovarian lesions were detected. Six patients (67%) had unilateral lesions and left ovary (67%) was most commonly involved in unilateral lesions. Most of the ovarian metastases were predominantly cystic in nature (44%), followed by mixed (34%) and solid (22%). Median SUVmax (interquartile range) for ovarian metastases was 5.9 (7.1) g/dL. All nine patients had imaging evidence of distant metastases (besides ovarian) at the time of presentation.

*Conclusion:* Ovarian metastases are usually unilateral and cystic in attenuation.

#### A009. Lymphoma Staging Using the Ann Arbor and Lugano Systems of Classification Evaluating Concordance

**R. Hussain[1], S. Nousheen[1], M. Ilyas[2]**
*[1]Jinnah Postgraduate Medical Center, Pakistan*
*[2]United Medical and Dental College, Pakistan*

*Aims:* New and more advanced regimens of treatments for the lymphomas have made the malignancy amendable to treatment. Treatment strategy, however, is highly dependent on the clinical stage of the disease and how the regimen given is seen to respond in any particular patient with adjustments to the same being made accordingly. The classification system for the staging of Hodgkin's lymphoma patients was originally established in 1971. It utilized computed tomography findings to evaluate and stage the disease in both the initial and post-treatment stages. The findings of this modality were, however, limited to its anatomic evaluation with no functional information being offered. In 2014, the Lugano classification was established. This system incorporated $^{18}$F-fluorodeoxyglucose-positron emission tomography/computed tomography as a means for objectively assessing the disease status with the advantage of the real-time functional information being incorporated. This allowed for a true assessment of the disease status, especially after treatment institution. To evaluate the utility of the Lugano against the Ann Arbor classification, we prospectively followed patients with lymphoma referred to our institution for initial staging, interim treatment, or end-of-treatment scanning.

*Methods:* This is ongoing research. All patients with lymphoma that were referred to our institution for scanning for initial baseline staging, interim treatment response, or end-of-treatment evaluation were included in our study. Whole body imaging from vertex to mid-thighs was acquired and analyzed.

*Results:* One-hundred eleven patients with a median age of 41.6 years (range: 10–75 years) were included in this prospective study. Ninety-five patients were included from the time of their baseline scans and 16 were followed from their first chemotherapy institution. The scans included were up to the post-third chemotherapy evaluation in some patients. A total of 151 scans, so far, have been included (this is an ongoing study). The Ann Arbor staging matched the Lugano stage in 128 scans (84.7%). Six scans were upstaged whereas 17 scans were downstaged using the Lugano classification.

*Conclusion:* The results of our study indicate a high concordance rate between the Lugano and Ann Arbor staging; however, the Lugano system has a decided advantage to offer to the staging of the disease status, especially in the post-chemotherapy follow-up of the patients.

### A010. Skeletal Muscle Uptake of $^{18}$FDG PET-CT: To Be or Not to Be? A Pictorial Essay

**R. Hussain[1], S. Nousheen[1], M. Ilyas[2]**
[1]*Jinnah Postgraduate Medical Center, Pakistan*
[2]*United Medical and Dental College, Pakistan*

*Introduction:* Skeletal muscle metastases (SMM) are a rare occurrence despite the fact that skeletal muscle comprises more than 50% of total body mass. When present, most are asymptomatic and are often found incidentally on imaging. Muscle resistance to both primary and metastatic malignancies is well-known, but its causes are still unclear. The factors preventing tumors from growing are believed to be connected with the ability of muscles to metabolize lactic acid, which selectively inhibits the proliferation of tumor cells in vitro and in vivo, inhibition by adenosine, or mechanical destruction of cancer cells within the microvasculature. Although considered rare, SMMs are suggested to occur more frequently than it is usually recognized. This may be a result of the improvement in quality and availability of imaging modalities, particularly the increase in utilization of fluorodeoxyglucose F18 positron emission tomography/computed tomography (18F-FDG PET/CT) in routine staging and follow-up of patients with different tumors.

*Aims and Objective:* Prime objective was to demonstrate uptake of $^{18}$FDG in the muscles on the oncological scanning. Secondary objective was to understand and classify the causes for these uptakes and help educate the various pathologies and present pictorial presentation for the same methods and materials: The PET/CT database from our institution was reviewed retro as well as prospectively from March 2020 to February 2023 for muscle metastasis from patients sent for oncological scanning with pathologically proven malignancies. The patients with SMM were reviewed and included in this pictorial case series study.

*Results:* Given as a pictorial presentation.

*Discussion:* The normal $^{18}$FDG uptake by the muscle is relatively mild and homogenous. The reason for this is the use of fatty acid oxidation by the muscles during period of rest. With the release of insulin into the circulation, as a result of food intake or exercise, there is a translocation of the GLUT-4 receptor from the cytosol to the plasma membrane. This results in uptake of $^{18}$FDG (an analogue of glucose) into the cell. Hence, a normal physiological uptake on scan is seen postprandial, with endo or exogenous insulin release or use/activity of muscle or muscle groups. This may also include stress-induced muscle tension spastic paresis, hyperventilation, and activities such as talking, chewing, and so forth. These uptakes are usually mild-to-moderate and symmetrical. Adequate patient education and preparation can help in reducing these uptakes. Pathological uptakes by the muscle may be benign (infective / inflammatory) or malignant. The benign uptakes may be due to a primary infective involvement or secondary as a sequel to surgery/radiation therapy. The skeletal musculature is a very well perfused organ; fortunately, however, as a result of increased lactic acid production during exertion, there is limited response of the blood vessels to the angiogenic stimuli of tumor deposit, rendering it relatively safe for secondaries. Despite this, muscle metastasis is still observed that may be asymptomatic (discovered on surveillance scanning), or cause local pain. Neoplasms involving the muscles can be either primary (benign/malignant) or secondary. Among the primaries that may involve the muscles, the more common seen in our set-up have been the sarcomas.

*Conclusion:* Skeletal muscle uptake on an $^{18}$FDG scan can be either physiological or pathological. Proper patient preparation and knowledge of the normal physiologic variants of FDG uptake in the skeletal muscles are essential for differentiating physiologic from pathologic conditions and in giving a final decision on the uptake for further management.

### A011. Assessing Vertebral Fractures with Bone Mineral Density Performed for Routine Diagnosis of Osteoporosis: Is There a Value Addition?

**R. Hussain[1], A. Ahmed[2], M. Ilyas[3]**
[1]*Jinnah Postgraduate Medical Center, Pakistan*
[2]*Zia-ud-Din Hospital, Karachi, Pakistan*
[3]*United Medical and Dental College, Pakistan*

*Background:* The dual energy X-ray absorptiometry (DXA) is a gold standard for the assessment of the bone mineral density and is also the only validated technology that uses the BMD input for the World Health Organization fracture risk assessment algorithm, FRAX. Identification of previously unrecognized vertebral fractures with VFA has the tendency to change diagnostic classification, fracture risk assessment, and decisions regarding treatment with alteration of management of the patient with reduction of morbidities. The rationale of this cross-sectional study is to seek and report the advantage of VFA with BMD (as reported by others) in our current settings and offer an algorithm for the assessment of osteoporosis. Acquisitions for the BMD and VFA are being done and reported with standard Hologic reference database for Caucasian males/females. After the routine BMD, VFA is performed in the same position with the C-arm of the machine moving to lateral position. After acquisitions, machine software is used to generate the BMD as well the VFA report and a comprehensive report stating the bone mineral condition as well as vertebral fracture assessment is made and reported. This study uses descriptive statistics only. Quantitative data includes age, expressed as mean $\pm$ standard deviation. Frequencies and percentages are being calculated for qualitative data such as gender and findings of osteopenia or osteoporosis (Yes/No). The subgroup comparisons are based on students $t$-test with a $p$-value of 0.0 5 as cut-off.

*Results:* In process; to be concluded when presented.

*Conclusion:* In process; to be concluded when presented.

#### A012. Esophageal Transit Scintigraphy versus Barium Swallow, Complementary Diagnostic Method for Examining the Esophagus

M. Bozinovic[1]
[1]Military Hospital Novi Sad, Serbia

Barium swallow is a dedicated test of the pharynx, esophagus, and proximal stomach, and may be performed as a single or double contrast study. Main indications for a barium swallow including high or low dysphagia, GERD, assessment of hiatus hernia, generalized epigastric pain, assessment of fistula, and persistent vomiting. Main contraindications are suspected perforation, postoperative assessment for leak and tracheoesophageal fistula. Esophageal transit scintigraphy has been used to screen symptomatic patients before considering more invasive procedures, to diagnose or exclude esophageal motility disorders. Widely used radiopharmaceutical is technetium-99m sulfur colloid, 200 to 300µCi (7.4–11.1MBq) dispersed in a small volume of clear liquid. Main advantage in this study is quantification. TACs can be derived for the entire esophagus or selected regions (proximal, medium, and distal part). Esophageal transit can be quantified by calculating a transit time (abnormal $>$15 seconds) or the percent residual activity in the esophagus (abnormal $>$20%). The esophageal transit study has high sensitivity for the detection of achalasia, but lower sensitivity for other disorders. In clinical practice, it is best to compare both conditions to avoid manometry.

### Thyroid

#### A013. A Study on the Development of Cancer in Hyperthyroid Patients Treated with Radioactive Iodine at Sultan Qaboos University Hospital

J. Al-Wardi[1], A. Alfutaisi[1]
[1]College of Health and Medical Sciences, Sultan Qaboos University, Oman

*Background:* Radioactive iodine I-131 (RAI) therapy is one of the methods to treat hyperthyroidism that raises the concern of risk of cancer development. To the best of our knowledge, no studies have been conducted on the incidence of cancer development in the Omani patient treated with RAI. This study aims to establish a relationship between the emergence of cancer with the RAI therapy in the hyperthyroid patients treated for hyperthyroidism in Sultan Qaboos University Hospital.

*Methods:* This retrospective cohort study was conducted on the data of 958 patients diagnosed with grave disease, thyroid toxic nodular goiter, or benign toxic neoplasm of thyroid and treated with RAI from January 2007 to January 2020. The data was collected using SQUH TrakCare system. The data was analyzed using IBM SPSS, ver. 26.0, New York, United States. The result was evaluated with the use of Z-test and a $p$-value of less than 0.05 will be considered significant.

*Results:* Eight out of 958 patients (0.8%) showed the emergence of cancer ($p = 0.078$). None of the patients who received two doses of RAI showed any development of cancer.

*Conclusion:* We have observed that RAI treatment is safe for the treatment of hyperthyroidism. There is no significant risk associated with the use of RAI in hyperthyroid patients.

#### A014. Differentiated Thyroid Carcinoma in Childhood and Adolescence

A. Mhiri[1], I. Slim[1]
[1]Salah Azaiez Institut, Tunis, Tunisia

*Introduction:* Thyroid cancer in children and adolescents is rare and has a good prognosis, but not infrequently present with lymph node and/or lung metastasis. It is usually a major concern for physicians, patients, and parents.

*Methods:* Our study included 25 patients thyroidectomized, aged between 5 and 17 years. All these patients have received radioiodine treatment and underwent a post-therapeutic whole-body scan, and serum thyroglobulin (Tg).

*Results:* There were 14 females and 11 males. The predominant histology was papillary carcinoma. Pulmonary metastases were detected in all of patients, detected only on the radioiodine scan. Therapeutic efficacy was observed after three cures of 131I in 14 patients and after two cures in 6 patients. In five cases, lymph node recurrence was revealed by concomitant reascension of Tg with occurrence of laterocervical foci on 131I imaging.

*Conclusion:* Differentiated thyroid cancer in child and adolescent is characterized by local aggressiveness and a higher frequency of lung metastasis than in the adult. The prognosis is usually very good with surgery that is the mainstay of treatment, followed by radioiodine therapy and hormonal thyroid suppression.

#### A015. Incidence of Second Primary Malignancies following Thyroid Cancer Treatment with Radioactive Iodine

A. Alhabsi[1], A. Alfataisi[1]
[1]Sultan Qaboos University, Oman

*Background:* Thyroid cancer is the most common endocrine malignancy, with an increasing incidence globally and in Oman. The standard treatment for differentiated thyroid cancer (DTC) involves radioactive iodine (RAI). However, previous studies have suggested that RAI treatment may increase the risk of second primary malignancies (SPM). Despite the high incidence of thyroid cancer in Oman, to our knowledge, there are no published reports on the association between RAI treatment and the risk of SPM in Oman.

*Objective:* There is a lot of debate about the possibility of developing SPM in DTC patients after treatment with RAI. This research aimed to evaluate the incidence and estimate the risk of SPM in thyroid cancer patients treated with RAI.

*Methods:* A retrospective cohort study was conducted at Sultan Qaboos University Hospital (SQUH) for 500 DTC patients who received RAI treatment between January 2007 and December 2017. We collected patients' information, including gender, age at diagnosis, thyroid cancer subtypes, site of SPM, cumulative RAI doses, and follow-up period. Descriptive statistics and logistic regression were used to analyze the data. SPM was defined as a new malignancy diagnosed at least 1 year after the first RAI dose.

*Results:* The mean follow-up period was $9.5 \pm 3$ years (range: 5.1–15.8). During this period, four patients (0.8%) developed SPMs, all with the papillary subtype. The sites of the SPMs were the colon, bladder, breast, and liver. We found age at diagnosis to be a significant predictor of the occurrence of SPMs ($p = 0.02$).

*Conclusion:* The incidence of SPMs in patients with thyroid cancer treated with RAI is low, and age at diagnosis was found to be the only significant predictor of SPM occurrence. These findings suggest that RAI treatment is safe and does not significantly increase the risk of developing SPM.

#### A016. Solitary Bone Metastasis of Hurthle Cell Thyroid Cancer with TENIS Syndrome: A Clinical Case

T. Geliashvili[1], A. Li[1], A. Parnas[1], O. Baranova[1], V. Ilyakov[1]
[1]*N. N. Blokhin National Medical Research Center of Oncology, Russia*

A significant role in improving the prognosis of differentiated thyroid cancer (DTC) in the presence of bone metastases is determined by early diagnosis of metastases, timely and correctly selected treatment tactics for the patient. During dynamic follow-up of patients with DTC after combined treatment (thyroidectomy with RAI therapy) determination of oncomarkers (serum thyroglobulin and antibodies to thyroglobulin) and ultrasound diagnostic of the neck, scanning with radioactive iodine (RAI). In some cases, patients have TENIS syndrome (thyroglobulin elevated negative iodine scintigraphy, hereinafter TENIS-syndrome), characterized by high serum thyroglobulin level in blood and absence of RAI were performed iodine accumulation on post-therapeutic scintigraphy. According to the research studies, positron emission tomography/computed tomography (PET/CT) with fluorodeoxyglucose F18 ($^{18}$F-FDG) has high sensitivity and specificity (89 and 72%, respectively) in visualization of metastatic radioiodine refractory foci in TENIS syndrome. This article presents a clinical case of a patient, a 52-year-old woman, with Hurtle cell thyroid cancer (pT3aN0M0, stage I) with established TENIS syndrome. Thyroidectomy was performed in September 2019 and radioiodine therapy was performed in January 2022 due to suspected disease progression given high thyroglobulin levels. Given the absence of pathologic accumulation of I-131 according to post-therapy radioiodine scanning, PET/CT with $^{18}$F-FDG was performed, which revealed a solitary metastasis in the left iliac bone (41x35x42 mm with maximum standardized uptake value: 17.25). In November 2022, radical treatment of the solitary bone metastasis was performed in the scope of resection of the left iliac bone with reconstructive-plastic component. According to the data of control examinations in June 2023, the patient has a complete biochemical and radiologic remission of the disease. This clinical case confirms the literature data on the aggressiveness of Hurtle cell thyroid cancer and the low response of bone metastases to RAI therapy; the significant role of $^{18}$F-FDG PET/CT in the search for metastatic foci in TENIS syndrome and the adoption of optimal treatment tactics.

#### A017. Safety and Efficacy of High Iodine 131 Therapy in Pulmonary Metastatic Papillary Thyroid Carcinoma in Children

H. Hammami[1], W. ElAjmi[1], A. Sellem[1]
[1]*HMPIT, Tunisia*

*Introduction:* Differentiated thyroid carcinoma is rare in childhood. It is frequently discovered in pulmonary metastatic stage, due to its diffuse character the treatment relies exclusively on iodine 131.

*Case Report:* We present a clinical case to illustrate the safety and efficacy of repetitive high dose of iodine 131 therapy in diffuse pulmonary metastatic thyroid carcinoma in a 6-year-old girl. The treatment consisted of a repeated iodine 131 cures every 4 to 6 months till complete remission. The total cumulative dose reached 24 GBq (650 mCi) after seven iodine 131 cures delivered in 27 months. After 15 years fellow-up based on thyroglobulin dosage, ultrasound, and iodine scans, no recurrence has occurred. Repeated respiratory functional exploration showed no lung damage secondary to high delivered doses. The patient was considered in total remission with no adverse effect of the repeated high iodine 131 therapy.

*Conclusion:* Iodine 131 therapy is the treatment of choice of pulmonary metastases with more than 80% efficacy. Spacing the cures decreases the risk of pulmonary fibrosis.

#### A018. The Role of Immunity Status Based on Lymphocyte Monocyte Ratio (LMR) in Treatments Response Low-risk Differentiated Thyroid Carcinoma Patients with High Antithyroglobulin Antibodies Pre-I-131 Therapy

J. Imaniar[1], B. Hidayat[2], E. Affandi Soeriadi[2], H. Budiawan[2]
[1]*Dharmais Cancer Hospital - National Cancer Center Indonesia, Indonesia*
[2]*Department of Nuclear Medicine and Molecular Imaging Dr. Hasan Sadikin General Hospital/Faculty of Medicine Universitas Padjadjaran Bandung Indonesia, Indonesia*

*Introduction:* Patients with differentiated thyroid carcinoma (DTC) after total thyroidectomy with high serum levels of anti-thyroglobulin (anti-Tg) antibodies before I-131 therapy will make it difficult to predict the success of therapy, especially in low-risk patients. So, it is necessary to look for other indicators that do not depend on Tg. Inflammation plays a key role in the initiation, promotion, and progression of cancer, by mediating the interaction of the immune response, influencing the prognosis and response to therapy. Some experts suggest that the lymphocyte-monocyte ratio (LMR) can indirectly reflect the immune status of the host and serve as a predictor of the prognosis of various malignancies.

*Methods:* In this observational retrospective single-center study, we reviewed all patients with histologically proven well-differentiated thyroid carcinoma with high pre-therapy serum anti-Tg antibody levels who underwent first I-131 at the Department of Nuclear Medicine, Dr. Hasan Sadikin Hospital Bandung between 2016 and December 2020. As immunity status based on LMR and low risk stratification. Low risk stratification criteria were determined by ATA 2015. Early treatment response is determined by the presence or absence of residual activity in the thyroid bed 6 months after radioiodine therapy. Logistic regression analysis was performed to assess the association.

*Results:* A total of 62 subjects of low-risk DTC patients with high pretherapy serum anti-Tg antibody levels were investigated. The median (min;max) LMR in the successful treatment group was 6.5 (2.82;112.9) and the unsuccessful treatment group was 4.2 (1.8;10.3), $p = 0.001$. By controlling the confounding variable, Tg. Successful response to the first I-131 therapy increased to 0,652 for every 1 unit increase in the LMR variable. Odds ratio value = 1.919 (95% confidence interval: 1.264–2.911; $p = 0.002$).

*Conclusion:* Patients with low-risk DTC after total thyroidectomy, who had high pretherapy serum anti-Tg antibody levels, high LMR were associated with higher success of the first I-131 therapy.

### Prostate

#### A019. Role of PSMA-PET/CT in Cancer Prostate: Initial Staging, Monitoring the Therapeutic Responses, Biochemical Recurrence and as Selective Tool for the Radioligand Therapy

Y. Atteya Mansour El Fiky[1], Y. Omar Heussin[2]
[1]*Faculty of Medicine-Helwan University, Cairo, Egypt*
[2]*Misr Radiology Center, Cairo, Egypt*

*Introduction:* The prostate cancer is one of the most common cancers among men. The overexpression of the prostate-specific membrane antigen (PSMA) in prostate cancer helps in many ways for the detection of the primary as well as the metastatic lesions. Recently in nuclear medicine, targeting the PSMA has been used for the diagnostic and therapeutic purposes with the evolving role of PSMA-positron emission tomography-computed tomography (PET/ CT) using the PSMA targeted imaging radiotracer agents as gallium-68. It is approved by Food and Drug Administration in 2020 is very important in early detection, initial staging, biochemical recurrence, monitoring the therapeutic responses as well as the rising role as selective tool used for the eligibility criteria in patients suitable for radioligand therapy (RLT).

*Methods:* This is a retrospective study that included 50 clinically or pathologically diagnosed patients as having prostatic cancer. This study was performed during the period between January 2021 and September 2023.

*Results:* The results from the present study and previous studies provide consistent evidence that the using of PSMA-PET/CT as noninvasive imaging tool can aid healthcare providers in diagnosis and assessing prostate cancer.

*Conclusion:* The PSMA-PET/CT is an evolving imaging modality and game changer with marked impact on patient management, playing an important role in changing the planes of management for each patient starting from the primary diagnosis, staging with metastatic disease detection, the post-therapeutic follow-up assessment, the biochemical recurrence till being currently used as major selective tool assessing the patient preparing for the lutetium PSMA RLT.

### A020. A Case of Unilateral Decreased PSMA Ligand Uptake in the Kidney Suggests Its Potential Use as a Functional Kidney Imaging

**S. Chokyakorn[1], C. Buakhao[2], N. Bunnag[2], T. Thongsricome[3], S. Vachatimanont[4]**
*[1]Nuclear Medicine Unit, King Chulalongkorn Memorial Hospital and Faculty of Medicine, Chulalongkorn University, Thailand*
*[2]Nuclear Medicine, Faculty of Medicine, Chulalongkorn University and Phramongkutklao College of Medicine, Thailand*
*[3]Diagnostic Radiology Unit, King Chulalongkorn Memorial Hospital and Faculty of Medicine, Chulalongkorn University, Thailand*
*[4]Nuclear Medicine Unit, Physiology Department and Nephrology Division, Faculty of Medicine, Chulalongkorn University, Thailand*
*Chokyakorn, S, King Chulalongkorn Memorial Hospital and Faculty of Medicine, Chulalongkorn University, Thailand*

*Introduction:* Although prostate-specific membrane antigen (PSMA) ligand imaging was initially developed for prostate cancer imaging, there has been increasing evidence of its potential use in kidney function assessment.

*Methods:* We present a case of a man in his eighties who has underlying of renal artery stenosis (RAS) and prostate cancer. He was sent for a gallium-68 PSMA-11 PET/CT for the restaging of prostate cancer. Incidentally, a unilateral decrease in PSMA uptake in the kidney of the patient that may correspond to his RAS was detected.

*Results:* The patient presented with an elevated serum PSA level of 18.1 ng/mL during ongoing hormonal therapy for his prostate cancer. His right RAS had been diagnosed after he presented with hypokalemia, difficult-to-control hypertension, and progression of chronic kidney disease to stage 3b. The PET/CT scan revealed a decreased PSMA avidity in the right kidney (right kidney maximum standard uptake value [SUVmax] = 5.06, SUVmean = 2.70; left kidney SUVmax = 38.9, SUVmean = 25.3) along with right hydroureter and right hydronephrosis. The decrease in PSMA ligand uptake could be attributed to either obstructive uropathy or underlying renal artery stenosis. Regardless of the cause, it supports the suggestion that PSMA ligand uptake corresponds to kidney function.

*Conclusion:* There have been several reports highlighting the correlation between PSMA ligand uptake and the kidney function, which suggest a potential novel application of PSMA ligand imaging for functional kidney imaging comparable to technetium-99m dimercaptosuccinic acid (Tc-99m DMSA). Our case supports this potential, but further research is necessary before considering the widespread adoption of PSMA ligand as an alternative to Tc-99m DMSA.

### A021. Samarium-153 Treatment of Bone Pain in Metastatic Prostate Cancer: About 80 Cases

**A. Mhiri[1], H. Hammami[2], I. Slim[1]**
*[1]Department of Nuclear Medicine, Salah Azaiez Institut, Tunis, Tunisia*
*[2]Department of Nuclear Medicine, Military Hospital, Tunis, Tunisia*

*Introduction:* Painful bone metastases are common in advanced prostate cancer. Samarium 153-ethylenediamine-tetramethylenephosphonic acid (153Sm-EDTMP) is a β-particles emitter that concentrates in areas of enhanced osteoblastic activity and used for palliate pain from bone metastases. The aim of our study was to assess the efficacy of (153)Sm-EDTMP therapy.

*Methods:* Eighty patients with metastatic prostate cancer received a single bolus infusion of 153Sm. Mean applied dosage was 37 MBq/kg of the patient's body weight. All patients had painful bone metastases to more than one anatomical region. Karnofsky performance status, pain score (numerical rating scale), analgesic score (WHO) and blood count were evaluated before, and 1 and 3 months after the treatment.

*Results:* We observed a positive response in 87% of the cases: complete in 36% of the cases. Responses observed after multiple administrations show that cures could be repeated with similar effects comparable to those of the first cure. Therapeutic efficiency is at least equivalent to those of the other therapeutic means, with nearly non-existent secondary effects. The only toxicity observed was of hematological order; it was mild and transient (grade 1 or 2) with a complete recuperation at the end of 8 weeks. Besides, the effect on the pain came with an improvement of the quality of life of the patients treaties.

*Conclusion:* Our study shows both the efficacy and the safety of Sm-153 in repeated treatments for metastatic bone pain in patients with prostate cancer suffering from painful bone metastases . Its administration offers clinical relevant pain relief with tolerable hematological toxicity and then enjoys a better quality of life, waiting the use of theranostics agents.

### A022. Unveiling the Power of PET-PSMA in Initial Staging of Prostate Carcinoma: KHCC Experience Essenced from PROCA Study

**A. Al-Ibraheem[1], D. Al-Adhami[1], F. Istatieh[1], A. Abdlkadir[1], E. Lobato[2]**
*[1]King Hussein Cancer Center (KHCC), Jordan*
*[2]International Atomic Energy Agency (IAEA), Austria*

*Introduction:* The aim of this retrospective study was to investigate the effectiveness of positron emission tomography (PET) and prostate-specific membrane antigen (PSMA) imaging in comparison to computed tomography (CT), magnetic resonance imaging (MRI), and skeletal scintigraphy for the initial staging of patients recently diagnosed with intermediate- and high-risk local prostate carcinoma.

*Methods:* In a retrospective study, 202 patients recently diagnosed with prostate carcinoma were initially evaluated using a multimodality approach. This approach involves initial assessment through the utilization of gallium-68 ($^{68}$Ga)-PSMA PET/CT, MRI, CT, and skeletal scintigraphy. Patients were exclusively enrolled if they were categorized as high- or intermediate-risk. A comparison of the sensitivity and specificity of these modalities was conducted, with established histology post-prostatectomy serving as the reference standard.

*Results:* A total of 202 patients with intermediate and high-risk prostate carcinoma were retrospectively enrolled. $^{68}$Ga-PSMA PET/CT exhibited superior performance over other modalities, demonstrating statistical significance ($p <$ 0.05, each). $^{68}$Ga-PSMA PET/CT had comparable accuracy to MRI in primary disease detection but a higher accuracy for nodal disease (97.1% versus 82%, $p = 0.03$). It also outperformed CT scans in nodal detection (97.1 vs. 73.8%), extrapelvic lymph nodes (100 vs. 69%), and bone lesions via skeletal scintigraphy (100 vs. 60%). Furthermore, the reliance on the staging results retrieved from $^{68}$Ga-PSMA PET/CT changed the management scheme for 97 patients (48%).

*Conclusion:* $^{68}$Ga-PSMA PET/CT is an invaluable imaging tool for patients with intermediate and high-risk prostate carcinoma. This novel imaging approach outperformed all other imaging modalities in the assessment of local, locoregional, and distant disease spread, with a significant impact on the management plan.

#### A023. Impressive Response to 177Lu-PSMA-617 Therapy in a Patient with Metastatic Castration-Resistant Prostate Cancer Refractory to Apalutamide, Docetaxel, and Metastasis-Directed Therapy

**T. Geliashvili[1], A. Li[1], A. Pronin[1], A. Krylov[1]**
*[1]N. N. Blokhin National Medical Research Center of Oncology, Russia*

Prostate-specific membrane antigen (PSMA) therapy has already established itself as a highly effective targeting therapy based on the theranostic concept of treat what we see and see what we treat. Here we describe an illustrative case of the efficacy and promise of this therapy in metastatic castration-resistant prostate cancer (mCRPC). The patient is an 84-year-old male diagnosed with prostate cancer cT3aN1M0, stage IV. For locally advanced disease external beam radiotherapy to the prostate gland and seminal vesicles and regional lymph nodes and constant androgen-deprivation therapy (ADT) during 28 months. The castration-resistant stage of the disease is further diagnosed—multiple metastases to bone and presacral lymph nodes are detected when prostate-specific antigen (PSA) is elevated on positron emission tomography/computed tomography (PET/CT) with PSMA. Three lines of mCRPC therapy were sequentially administered: metastasis-directed therapy (MDT) by stereotactic radiotherapy to bone foci in combination with ongoing ADT; apalutamide in combination with MDT on bone foci and lymph nodes plus ongoing ADT; five courses of docetaxel chemotherapy with marked hematologic toxicity. The response to each of these lines of therapy was progression within 5 to 6 months. From December 2022 to May 2023, PSMA-targeted therapy with 177Lu-PSMA-17 (4 courses at 6–8 weeks intervals, mean activities of 7.6 GBq) was given as fourth-line therapy for mCRPC. Treatment was terminated due to the development of complete radiologic response and the absence of metabolic substrate for continuation of therapy according to the theranostic concept. PSA response: decrease in total PSA deceased from 17 (12/2022) to 0.011 ng/mL (06/2023). Except for xerostomia grade 1, no other toxic reactions were noted. As of December 2023, patient status was as follows: ECOG—0, PSA total—0.018 ng/mL, remission according to PET/CT with PSMA, time without progression 12 months. In a patient with aggressive and refractory to standard drug therapy for mCRPC, PSMA-targeted radioligand therapy with 177Lu-PSMA-17 was highly effective and low toxicity.

#### A024. PSMA PET/CT Interpretation Pitfalls

**M. Elkanawaty[1]**
*[1]MISR Radiology Center and MOHP, Egypt*

*Introduction:* The prostate-specific membrane antigen positron emission tomography/computed tomography (PSMA PET/CT) is increasingly used in the evaluation of prostate cancer. However, PSMA is expressed physiologically in nonprostatic tissues, as well as in other pathological conditions.

*Methods:* This is an educational case-based approach aiming to illustrate the different pitfalls in PSMA PET/CT interpretation, highlighting the variation in physiological distribution of PSMA activity and uptake in various benign and neoplastic conditions that may be misinterpreted as metastatic prostatic disease. This is to increase the awareness and to aid in more accurate interpretation of the PSMA PET/CT.

*Results:* To differentiate 1) Structures with physiological PSMA uptake which may be mistaken as metastatic lesions, like celiac ganglia, etc.; 2) Non metastatic active lesions with PSMA uptake like Paget's disease of the bone or other primaries as hepatocellular carcinoma or renal cell carcinoma, which can be mistaken as metastatic lesion; 3) False-positive PSMA uptake in nonspecific bony lesions, when using $^{18}$F-PSMA tracer; 4) To understand the difference between $^{18}$F and gallium-68 ($^{68}$Ga) PSMA, and pattern of excretion of each tracer as 18F has predominant hepatobiliary excretion, unlike $^{68}$Ga which has predominant renal excretion.

*Conclusion:* This work illustrates different pitfalls in PSMA PET/CT interpretation, highlighting the variation in physiological distribution of PSMA activity and uptake in various benign and neoplastic conditions to prevent misinterpretation of such conditions as metastatic prostatic disease.

#### A025. $^{177}$Lu-PSMA Radioligand Therapy for Metastatic Castration-Resistant Prostate Cancer: Kuwait experience

**S. Usmani[1], S. Murad, *[1] A. Esmail[1], F. Al Kandari[1], I. Alrekhais[1], S. N. Khalaf[1], R. Rasheed[1]**
*[1]Department of Nuclear Medicine, Kuwait Cancer Control Centre, Kuwait*

*Presenting Author

*Purpose:* Lutetium-177 prostate-specific membrane antigen (177Lu-PSMA) radioligand therapy (RLT) is an emerging treatment in metastatic castration-resistant prostate cancer (mCRPC). The purpose of this study is to present institutional experience of Kuwait, the effectiveness, efficacy, and safety data of 177Lu-PSMA RLT in mCRPC.

*Methods:* Twenty-one consecutive patients of mean age of $71 \pm 10.3$years with mCRPC received two to three cycles of 177Lu-PSMA after exhaustion of approved therapy (hormonal therapy and/or taxane chemotherapy). Patients were treated with Ministry of Health, Kuwait approval (1159/2019). Eligibility criteria include positive gallium-68 ($^{68}$Ga)-PSMA PET uptake above or equal to liver activity. The 4 to 8 GBq with median activity of 7.0 GBq per cycle were administered with 8-week time interval between consecutive cycles.

The primary outcome measure of our study was to report the prostate-specific antigen (PSA) response to 177Lu-PSMA RLT. Both any PSA level and greater than 50% PSA decline were analyzed. The secondary outcome measures were objective, radiological response according to PERCIST and safety. Toxicity was categorized by the Common Terminology Criteria for Adverse Events (version 4.03).

*Results:* Of the 21 patients treated with median of two cycles of 177Lu-PSMA RLT (range: 2–5cycles) with mean dose of $7.0 \pm 1.02$GBq, PSA response rate of 50% PSA decline was seen in 15 of 21 patients (52%) and any level PSA decline in 67%. PSA progression was encountered in seven (33%) patients. $^{68}$Ga-PSMA PET/CT follow-up 2 months after the last injection showed complete response (20%), partial response (30%), stable disease (10%), and progressive disease in 40%. Additionally, pain relief in 81% and improved quality of life/ performance status in 62%, were observed. 177Lu-PSMA RLT has minimal adverse effects. No acute or severe adverse events were observed. No evident kidney toxicity and xerostomia was found in this study.

*Conclusion:* 177Lu-PSMA RLT is a safe promising agent with minimal adverse effects in the treatment of patients with mCRPC that has progressed after standard treatment. Improved performance status and biochemical response are seen in more than half of the patients. The results of this study indicate that 177Lu-PSMA should be used as standard of care in the treatment of mCRPC.

#### A026. $^{177}$Lu-PSMA-617 in the Treatment of Metastatic Castration-Resistant Prostate Cancer: First 5 years of Experience in Uruguay

**G. dos Santos[1], I. Cordero[1], A. Banchero[1], A. Quagliata[1], S. Rodriguez[1], J. Gambini[1], P. Duarte[1], O. Alonso[1]**
*[1]Centro Uruguayo de Imagenología Molecular (CUDIM) y Hospital de Clínicas, Universidad de la República, Uruguay*

*Aim:* Prostate cancer is the most frequent solid neoplasm in the world and the second leading cause of cancer death in our country. The aim of this study was to assess the therapeutic response and safety profile of lutetium-177 prostate-specific membrane antigen ($^{177}$Lu-PSMA) in the treatment of patients with metastatic castration-resistant prostate cancer (mCRPC) in Uruguay.

*Methods:* We retrospectively analyzed 25 patients (who received at least one new androgen-axis drug and 1 or 2 taxane regimens) with mCRPC at CUDIM in the period June 2017 to June 2023. All underwent radiotargeted therapy receiving doses of 177Lu-PSMA-617 (7.4GBq every 6 weeks) for at least three cycles. Serial positron emission tomography/ computed tomography (PET/CT) scans were performed with gallium-68 ($^{68}$Ga)-PSMA-11, [$^{18}$F]AIF-PSMA-11, or $^{18}$F-PSMA-1007 to confirm PSMA expression in metastatic lesions and evaluate response. Four patients underwent an additional fluorodeoxyglucose F18 ($^{18}$F-FDG) PET/CT scan, excluding FDG+/PSMA- patients.

*Results:* The uptake of PET PSMA tracers and $^{177}$Lu-PSMA-617 was similar in single-photon emission tomography and PET images. All four $^{18}$F-FDG PET/CT scans were negative (PSMA positive), thus suitable for treatment. Eighteen patients (72%) had lesion regression by PET-PSMA control imaging (positive response). The average overall survival was 14.7 months. Sixty-three percent of patients showed biochemical response (prostate-specific antigen decrease). In some cases, there was a decrease of 90%. None of the patients had serious adverse effects (xerostomia, nausea, fatigue, and thrombocytopenia) or had to discontinue treatment due to toxicity. Sixty-eight percent of patients reported sustained pain relief after treatment.

*Conclusion:* $^{177}$Lu-PSMA therapy is a promising option in the treatment of patients with mCRPC, whose survival and quality of life have not been improved despite the myriad treatments available. $^{68}$Ga-PSMA-11, [$^{18}$F]AIF-PSMA-11, and $^{18}$F-PSMA-1007 versus $^{177}$Lu-PSMA prove to be similar diagnostic-tandems. Experience in our environment shows a benefit in overall survival, lesions progression, and pain relief. Data was consistent with scientific evidence that determined the approval of this therapeutic line in our country.

#### A027. $^{177}$Lu-PSMA First Successful Therapy Performed in East Africa: A Case Report from Mulago National Referral Hospital, Kampala, Uganda

**J. S. Mugisha[1,2], A. K. R. Embati[2], S. Sibiya, M. Kibwika[2], M. Katumba[2], T. C. G. Moalosi, Z. Muyinda[2], I. A. Muramuzi[2]**
*[1]Mbarara University of Science and Technology/Mulago National Referral Hospital, Uganda*
*[2]Mulago National Referral Hospital, Uganda*

*Background:* Lutetium-177 prostate-specific membrane antigen ($^{177}$Lu-PSMA) radioligand therapy (RLT) is an important form of treatment for prostate cancer (PCa) patients. Its availability is predominantly in resource rich and adequately resourced and (equipment) established regions/ or countries unlike the east African region that is resource and equipment constrained.

*Case Presentation:* A 69-year-old man in 2015 with total prostate-specific antigen (TPSA)=75ng/mL was diagnosed with adenocarcinoma prostate, Gleason 7 (4+3) following prostate biopsy. On November 17, 2015, a technetium 99m-methyl diphosphonate bone scan (whole body) done showed no bone metastases. He then underwent a robotic radical prostatectomy with good recovery postsurgery. Histology reports of the postsurgical prostate tissue—adenocarcinoma prostate Gleason 9 (4+5) and circumferential margins showed involvement by tumor. He then had injection Zoladex and adjuvant external beam radical radiotherapy to the pelvis. Since 2016, TPSA had been stable below 0.2 ng/mL until November 2022 when TPSA had increased to 0.61 ng/mL, doubling in just 3 months in keeping with biochemical recurrence. A gallium-68 ($^{68}$Ga)-PSMA PET/CT scan performed showed $^{68}$Ga-PSMA avid PCa metastases in infra and supradiaphragmatic lymph nodes.

*Therapy and Imaging:* Following the PET/CT study and report in March 2023 as well as consideration of the therapy options, the patient was treated successfully with $^{177}$Lu-DOTA-iPSMA 9-months (delayed) later. The procedure was conducted in the nuclear medicine department of Mulago national referral hospital, Kampala, Uganda by a multidisciplinary team that comprised of Ugandans and a South African. Planar and single-photon emission tomography images post-therapy were obtained using Mediso Any scan at 6- and 24-hours' time points to evaluate $^{177}$Lu-DOTA-iPSMA uptake and for dosimetry purpose. The patient was discharged after the 24 hours imaging with a radiation of 4.0 µSv/hour at 1-m distance.

*Conclusion:* $^{177}$Lu-PSMA RLT is an important treatment option for PCa patients. However, its access is very limited in resource-limited/constrained areas. Our case is an excellent example of how RLT can be rolled out/made available to patients who qualify and need this form of treatment among PCa patients in resource-constrained regions like Uganda.

## Neuroendocrine

#### A028. Routine Early $^{68}$Ga-DOTATATE PET/CT Has Low Diagnostic Yield after Resection of Pancreatic Neuroendocrine Neoplasms

A. Singh[1], D. Chan[1], J. Samra[1], N. Pavlakis[1], A. Engel[1], C. Diakos[1], S. Clarke[1], A. Chou[1], E. Bernard[1], D. Bailey[1], P. Roach[1]
[1]*Royal North Shore Hospital, Australia*

*Introduction/Background:* Following resection of primary pancreatic neuroendocrine neoplasm (P-NEN), the reported median disease-free survival is more than or equal to 4 years. Gallium-68 ($^{68}$Ga)-DOTATATE PET/CT (DOTA-PET) is increasingly used for staging and restaging following surgical resection of P-NEN due to a higher sensitivity compared with cross-sectional imaging. We hypothesized that early DOTA-PET has a low yield in detecting recurrent/residual or distant disease in patients following curative-intent surgical resection of non-metastatic localized P-NEN.

*Aim:* The aim of this study was to analyze the diagnostic yield of DOTA-PET following curative-intent surgical resection of non-metastatic localized P-NEN.

*Methods:* Retrospective study (dual read) of patients undergoing DOTA-PET between 05/2011 and 03/2022; 0 to 16 months after curative-intent resection of P-NEN. Preoperative DOTA-PET and initial post-operative DOTA-PET were reviewed by an experienced nuclear medicine physician.

*Results:* Seventy-six P-NEN patients (median age, 64Y; 37% female) with World Health Organization tumor grade G1 ($n = 52$), G2 ($n = 18$) and G3 ($n = 3$) were included. Tumor grade was unavailable for three patients. The analysis demonstrated complete remission in 65/76 (85.5%) and inconclusive findings in 11/76 (14.5%) patients. There was no definitive evidence of Dota-avid residual/recurrent or distant metastases. On follow-up of these 11 patients, 9/11 had no recurrence, 1/11 developed liver metastases, and 1/11 was lost to follow-up.

*Conclusion:* $^{68}$Ga-DOTATATE PET/CT has low diagnostic yield when performed in the first 16 months after curative-intent resection of nonmetastatic localized P-NEN. There was no evidence of recurrence in 85.5%, and inconclusive findings in 14.5% patients. Despite advantages over cross-sectional imaging, DOTA-PET should be reserved for delayed restaging in this cohort of patients.

#### A029. Role of $^{68}$Ga-DOTA-NOC PET/CT in Detection of Primary Site in Patients with Metastatic Neuroendocrine Tumor of Unknown Origin: Experience from Pakistan

A. Saleem[1], A. Parveen[1], A. Shami[1]
[1]*Institute of Nuclear Medicine and Oncology, Lahore (INMOL), Pakistan*

**Keywords**

- neuroendocrine tumors (NETs)
- $^{68}$Ga-DOTA-NOC PET/CT
- maximal standardized uptake value (SUVmax)
- primary site
- metastatic NETs of unknown origin

*Introduction:* Neuroendocrine tumors (NETs) (historically called APUDomas) arise from neuroendocrine cells in the endocrine and central nervous systems. These are rare tumors accounting for 0.5% of all cancers. Gastrointestinal tract is the most common location and is responsible for two-thirds of NETs, followed by lung (one-third). However, quite a significant proportion present with metastases with unknown primary site. The advent of positron emission tomography/computed tomography (PET/CT) and specifically the $^{68}$Ge/$^{68}$Ga generator has revolutionized imaging of NET. The purpose of this study was to prospectively evaluate the role of $^{68}$Ga-DOTA-NOC PET/CT in detection of primary site in patients with metastatic NETs of unknown origin.

*Methods:* In this prospective, single-arm, single-institutional study, 38 patients with histologically proven metastatic NETs and unknown primary site on conventional imaging were recruited for the study. All patients underwent diagnostic $^{68}$Ga-DOTA-NOC PET/CT. Histopathology whenever possible and/or follow-up imaging were taken as reference standard. Maximal standardized uptake value of possible primary and metastatic sites was calculated.

*Results:* The highest number of patients presented with hepatic metastases, that is, 23 out of 38 patients (60%). $^{68}$Ga-DOTA-NOC PET/CT was able to identify the possible primary site in 20 out of these 38 patients (52%). Most common possible primary sites were small intestine (12) and pancreas (6) followed by lung (1) and rectum (1). Histopathology and follow-up imaging confirmed the primary site in 16 out of 20 patients (true positives 80%). Biopsy/follow-up imaging was not able to confirm the primary site in two patients (False positives 10%) and two patients were lost to follow-up. Among the 18 patients in whom $^{68}$Ga-DOTA-NOC PET/CT was not able to identify the possible primary site, follow-up imaging reflected the same in 16 patients (True negatives 88%), whereas follow-up imaging revealed possible primary sites in two patients, that is ileum and pancreas (false negatives 11%). The sensitivity and specificity of $^{68}$Ga-DOTA-NOC PET/CT came out to be 88 and 90%, respectively.

*Conclusion:* $^{68}$Ga-DOTA-NOC PET/CT is superior to conventional imaging modalities in detection of primary site in patients with metastatic NETs of unknown origin.

#### A030. Value of F-18 DOPA PET-CT Scan in Pheochromocytoma: First Experience in Indonesia

A. Dewi[1], J. Pribadi[1], Y. Tuti[1]
[1]*Dharmais Cancer Hospital, Indonesia*

*Introduction:* This is a case report of 22-year-old female with pheochromocytoma who underwent right adrenal resection.

*Methods:* Despite the surgical intervention, patient still in hypertensive condition, was referred for I-131 MIBG scan, which was positive for left femur metastasis. The patient then underwent radiation therapy for the metastasis, and her blood pressure returned to normal afterward. Another I-131 MIBG scan was conducted for postradiation therapy evaluation, still positive for the metastasis. The patient remained in good clinical condition and was referred for monitoring two years later. However, due to I-131 MIBG shortage in Indonesia, F-18 DOPA positron emission tomography-computed tomography (PET-CT) scan was performed, which showed stable disease.

*Result:* Pheochromocytoma is a rare catecholamine-producing neuroendocrine tumor that arises from adrenal medulla. Functional imaging plays important role in pheochromocytoma management. While many guidelines recommend somatostatin receptor PET-CT, FDG PET-CT, or MIBG Scan for evaluation, F-18 DOPA PET-CT scan has advantages for pheochromocytoma evaluation. F-18 DOPA specifically taken up by pheochromocytoma cells through binding with LAT1 then follows the metabolic pathways of L-DOPA. Unlike MIBG and other PET tracer, F-18 DOPA shows lack of significant uptake in the adrenal gland. F-18 DOPA also has fewer drug interaction compared with MIBG and DOTA-peptide.

*Conclusion:* Choosing the most relevant modality can be challenging. F-18 DOPA when available provides several advantages for pheochromocytoma evaluation.

## Miscellaneous

#### A031. Indonesian Health Transformation in Nuclear Medicine Services: Proctoring and QUANUM Implementation Programs

Y. Tuti[1], M. Abdulgani[1], N. Nurhuda[1], J. Imaniar[1], A. Dewi[1], R. Wigati[1]
[1]*Dharmais Cancer Hospital-National Cancer Center, Indonesia*

*Introduction:* The Ministry of Health of Indonesia is currently conducting a health system transformation, aiming to provide equal healthcare services across all hospitals in Indonesia, particularly focusing on cancer, heart, stroke, and uronephrology diseases. This health transformation program will also impact the expansion of nuclear medicine (NM) services in Indonesia, which currently limited to only 13 NM centers. It is planned to open new NM centers in 22 top referral hospitals and 38 major hospitals from 2022 to 2025. Dharmais Cancer Hospital, as the leader of cancer services for all hospitals in Indonesia, implements a proctoring and QUANUM programs. This is expected to standardize and enhance the quality of NM services throughout Indonesia.

*Methods:* Proctoring was conducted at 5 NM centers by the Dharmais Cancer Hospital NM team, consisting of NM physician, radiopharmacist, medical physicist, technologist and staff of building and equipment department, all experienced in NM services. This study reports the impact of proctoring results on new centers, those under construction, and existing NM service centers.

*Results:* Proctoring was performed at 5 NM centers, comprising three new centers, one center under construction, and one existing center. Subsequently, QUANUM program was conducted for two NM centers that had previously undergone internal audits. For the remaining three centers, QUANUM program awareness sessions were conducted. The NM team compiled a proctoring activity report. Findings from the proctoring activities at these five centers were reported, followed by recommendations and feedback to the hospital management.

*Conclusion:* Our study indicates that the proctoring and QUANUM programs have an overall positive impact on new, under-construction, and existing centers, enhancing the standard of NM services in Indonesia.

#### A032. Personalized Dosimetry Improves Progression-Free Survival after Selective Internal Radiation Therapy in Patients with Hepatocellular Carcinoma

W. Noordzij[1], D. Yigit[1], M. Veldhuizen[1], K. Akhundov[1], J. Van Sluis[1], S. Suiter[1]
[1]*UMCG, The Netherlands*

*Introduction:* Selective internal radiation therapy (SIRT) is an intra-arterial treatment used for patients with unresectable hepatocellular carcinoma (HCC) using either yttrium-90 labeled glass or resin microspheres. This study aims to compare the overall and progression-free survival of HCC patients treated with SIRT using standard (glass) and personalized (resin) dosimetry.

*Methods:* Data of consecutive uncomplicated treatments in our tertiary referral center were retrospectively analyzed: from November 2016 to October 2018 (glass) and June 2021 to November 2023 (resin). Patients were selected based on Child-Pugh A score, and treatment to only one lobe. Patients with other diseases than HCC and missing follow-up were excluded. Response to the treatment was determined on follow-up imaging (magnetic resonance imaging or computed tomography) at 3, 6, 9, and 12 months. Overall and progression-free survival were determined using the Kaplan–Meier estimate.

*Results:* Both glass and resin groups consisted of 15 patients. Glass patients were younger: median age 63 (range: 52–78) versus 70 (58–82) $p = 0.029$. No significant differences were found in other baseline characteristics. Median administered activity was 2,300 (270–7331) MBq in the glass group versus 1,500 (800–3280) MBq in the resin group, $p = 0.081$, whereas the median dose to the tumor was 300 (45–850) Gy (glass) versus 190 (105–420) Gy (resin), $p = 0.32$. Median time to progression was 193 days in the glass group, and not reached in the resin group, $p = 0.009$. At 12 months follow-up, 9 glass patients (60%) and 3 resin patients (20%) were deceased, resulting in a significant difference in overall survival (median overall survival 360 days vs. not-reached; $p = 0.03$).

*Conclusion:* HCC patients treated with glass microspheres using standard dosimetry show shorter progression-free and overall survival compared with those treated with resin microspheres using personalized dosimetry.

#### A033. Artificial Intelligence in Nuclear Medicine: Enhancing Precision, Efficiency, and Outcomes

Z. Chiragh[1]
[1]*ZAK-MI Consultancy, Pakistan*

Artificial intelligence (AI) is rapidly transforming nuclear medicine, offering unprecedented improvements in diagnostic accuracy, efficiency, and patient treatment outcomes. This article discusses the multifaceted impact of AI integration in nuclear medicine, highlighting its role in enhancing image acquisition, diagnostics, and treatment planning. With the ability to process extensive imaging data, AI provides real-time feedback and identifies disease indicators, significantly advancing diagnostic precision. This article explores the global trends in AI adoption in nuclear medicine, emphasizing the increasing demand and the resultant innovative solutions and collaborations among various stakeholders. Challenges in integrating AI, such as substantial initial costs, compliance with data privacy regulations, and the necessity for workforce training, are addressed. This article underscores the importance of the FUTURE-AI framework, which sets foundational principles for AI implementation in medical imaging. Anticipated advancements include sophisticated AI algorithms, integrations with augmented reality, and broad applications in nuclear medicine, all heralding a new era in precision medicine. Furthermore, this article examines the impact of AI on various stakeholders, including patients, physicians, and institutions. Patients benefit from personalized treatment plans and minimized side effects, while physicians enjoy advanced diagnostic tools and streamlined workflows. Institutions gain from enhanced competitiveness and improved patient satisfaction. Additionally, this article delves into the ethical considerations and the need for transparency and accountability in AI algorithms. In conclusion, the integration of AI in nuclear medicine is poised to revolutionize the field, improving resource allocations and patient outcomes. However, realizing its full potential requires ongoing research, development, and interdisciplinary collaboration. This advancement marks a significant shift toward precision medicine, with AI at the forefront, shaping the future of nuclear medicine.

**A034. Advancing Lung Cancer Diagnosis and Management: Integrating AI with PET/CT Imaging**

Z. Chiragh[1]
*[1]MI-ZAK Consultancy, Pakistan*

Lung cancer remains the most common and lethal cancer worldwide. Recent advancements in artificial intelligence (AI), particularly in deep learning (DL) and machine learning (ML), are revolutionizing its diagnosis and management. This synthesis explores the integration of AI with [$^{18}$F] fluorodeoxyglucose positron emission tomography/computed tomography ([$^{18}$F]FDG-PET/CT) in enhancing the detection and classification of lung cancer. One study demonstrated the use of a machine-learning-based algorithm to automatically classify adenocarcinoma, the most common lung cancer type, from other tumors using dynamic PET data. This algorithm achieved a high probability ($0.943 \pm 0.090$) for detecting adenocarcinoma, indicating its potential in improving diagnostic specificity and aiding in metastatic spread identification. Another publication highlighted the significance of radiomics and AI in nonsmall cell lung cancer, focusing on early detection, staging, and outcome prediction. Radiomics, a process of converting conventional images into mineable data, combined with AI algorithms, showed promise in enhancing the diagnostic performance of [18F]FDG-PET/CT. Furthermore, a study using a Lung Cancer Prediction Convolutional Neural Network trained on the National Lung Screening Trial data demonstrated its effectiveness in identifying benign lung nodules with high accuracy, potentially reducing unnecessary follow-ups and invasive procedures. This approach, which achieved a sensitivity of 99% and specificity of 22.1% in ruling out malignancy, underscores the utility of AI in lung cancer screening and risk prediction. In conclusion, the integration of AI with PET/CT imaging in lung cancer presents a paradigm shift in oncological diagnostics. By harnessing the power of radiomics, ML, and DL, it is possible to improve the accuracy of lung cancer diagnosis, tailor treatment strategies, and enhance patient outcomes.

**A035. A Survey on Incident Reporting and Learning System among Healthcare Workers in the Nuclear Medicine Department, Single-Institute Experience**

N. Marwat[1], S. Fatima[1]
*[1]AECH-NORI, Pakistan*

Incident reporting is promoted as a method for improving safety in critical industries and is now well established in healthcare facilities in many countries. One essential aspect to improve patient safety and reduce medical errors focuses on the need for healthcare organizations to promote a patient-safety culture, and to banish the blame and shame culture and "conspiracy of silence" traditional approaches within organizations when reacting to medical errors. Mature and well-established safety culture has known to be a common characteristic of high performing health care organizations. Safety culture is the product of individual and group values, attitudes, and perceptions that determine the commitment to an organization's health and safety management. This study aimed to measure knowledge and perception of existing incident reporting culture among workers of nuclear medicine department in a single hospital of located in the capital of Pakistan; Incident reporting is a major part of safety culture where whole nuclear medicine staff work together to create the safe and efficient clinical environment for patients, staff, and public. Incorporating incident reporting and learning into nuclear medicine practice have the potential to improve patient safety and increase the quality of care. Safety culture is a critical component of patient safety in any medical setup. Errors in nuclear medicine are not frequent, but one occurring can end up being critical (6). The survey results suggest that management commitment toward patient safety is important; reporting of incidents and especially reporting of near misses shall be encouraged; near misses can play a pivotal role in reducing future incidents. Policymakers and management shall build an environment that aims to improve communication within the unit and outside unit; elimination of disciplinary culture would serve to enhance the safety culture of the nuclear medicine department. The survey highlighted the aspects of patient safety culture that requires attention and needed discussion. The barriers and challenges that are abstaining from the staff from reporting errors were highlighted. In general, staff working in nuclear medicine feels positive toward safety culture as positive views were observed and building it to be more effective by reporting incidents and near misses. Frequency of reported events has to be increased. One need to pay attention and report about an event that was caught and corrected before affecting the patient. Events that have taken place but have no potential to harm the patient need to be reported. Near misses are more important than incident as these were caught before harming patient. The national reporting system can be developed where everyone can add their incidents and its analysis shall be open for all with anonymity. Anonymity is important as many organizations hesitate to report failures because of fear of bad publicity or patients getting information about the event and can use it in a lawsuit; staff from organizations will be more confident in reporting and the number of the event reported will increase after the anonymity aspect. The operation of national reporting and investigation system can raise awareness of the potential for things to go wrong and based on these experiences safety culture system can be improved.

**A036. Occupational Exposure of Workers in the Nuclear Medicine Department**

N. Marwat[1], S. Fatima[1]
*[1]AECH-NORI, Pakistan*

The present work was aimed to assess the radiation safety at nuclear medicine department being a work environment. Radiation exposure of the employees in the past 20 years and the effects of legislative changes in radiological protection were analyzed. Radiation workers at the nuclear medicine department were regularly and individually monitored using TLD and film badges. Annual doses of the employees of nuclear medicine department, registered in the period 2005 to 2016, were analyzed statistically. Technicians were found to be the largest exposed professional group, whereas radiopharmacist received the highest annual dose. Physicians received an average annual dose at the border detection methods. Ancillary staff occasionally received doses above the method detection limit (MDL). The average annual dose for all dosimetry records was 0.6 mSv, and that for dosimetry records equal and higher than MDL was 1.7 mSv. There was no case of an exceeded dose limit for a worker. Furthermore, improvement in radiological protection had a significant impact on the reduction in doses for the most exposed employees.

#### A037. The Possible Role of Combined [$^{18}$F]-Fluoroestradiol ($^{18}$F-FES) and $^{18}$F-FDG PET/CT Scans in Early Detection of Molecular Heterogeneity and Prognosis to Hormonal Treatment Response in Metastatic Estrogen Positive (ER+) Breast Cancer (BC) Patients: A Case Report

N. Rasulova[1], O. Ragab[1], S. Contu[1], E. Ahmed[1]
[1]*Al-Adan Hospital, Kuwait*

*Background:* F 18 fluoroestradiol (F-18 FES) is the first F-18 positron emission tomography/computed tomography (PET/CT) imaging agent indicated for the detection of estrogen receptor (ER)-positive lesions in patients with recurrent or metastatic breast cancer (BC), while the fluorodeoxyglucose F18 ($^{18}$F-FDG) can estimate the glucose metabolism and plays an important role in the management of BC patients.

*Aim:* The aim of this study was to estimate the role of combined $^{18}$F-FES and $^{18}$F-FDG PET/CT scans in detection of molecular heterogeneity, prognosis response to hormonal treatment, and close follow-up in metastatic ER BC patients.

*Methods:* A 28-year-old female with ER+ initially metastatic BC, after excessive treatment for 2 years with almost metabolically negative bone metastases and mildly FDG-avid primary lesion, being on Femara, Zoladex, and Xgeva, underwent $^{18}$F-FES and $^{18}$F-FDG scans after discovering a new axillary metastasis, followed by series $^{18}$F-FDG and $^{18}$F-NaF for close follow-up scans.

*Results:* $^{18}$F-FES and $^{18}$F-FDG scans showed matching FES/FDG-avid in the primary right breast lesion and axillary lymph nodes (maximum standardized uptake value [SUVmax] FES/FDG for primary lesion 6.33/1.89 and axillary lymph nodes 7.58/6.15) as well as FES negative/FDG-positive newly developed bone lesion in the head of left femur (FDG SUVmax 3.65), confirmed by NaF PET-CT scan. Patient refused to change the therapy and on close follow-up scans within 1 month FDG showed a disease progression at the primary site (FDG SUVmax raised to 2.45, on further follow-up scans to 7.5), axillary lymph nodes became multiple with (SUVmax 6.58), persistent hypermetabolic bone lesion (FDG SUVmax 4.02) and on NaF scan progression of the lesion in the head of the left femur as well as reactivation of the old metastases.

*Conclusion:* A single FES-negative/FDG-positive lesion may indicate appearance of early metabolic heterogeneity in metastatic BC and may play role in the prognosis and hormonal treatment response that can help to modify the treatment plan in ER+ BC patients receiving hormonal treatment.

#### A038. Noninvasive Treatment of Recalcitrant Keloids with Contact Brachytherapy Using Yttrium-90 Skin Patch and Its Effectiveness: A Pilot Study

S. Sundarraj[1], H. Nellaiyappar[1], N. Pandit[1], D. Nesamani[1]
[1]*JIPMER, India*

*Introduction:* Keloids are benign dermal fibroproliferative tumors. There are multiple treatment modalities for keloids in clinical practice with variable success rates. Some of the keloids may go unresponsive or recurred. These keloids are referred to as recalcitrant keloids. We have analyzed the effect of the yttrium (Y-90) skin patch on recalcitrant keloids in the Indian population. The primary objective was to determine the percent change in the thickness (depth) of the recalcitrant keloid every 2 months till 6 months post-therapy. The secondary objective was to determine the symptomatic relief post-therapy.

*Methods:* One mCi of Y-90 skin patch was applied locally on the lesion (sample size, $n = 28$) for 3 hours. Then the patients were followed up at 2 months, 4 months, and 6 months intervals. On each follow-up, visual assessment of the lesion, high-resolution ultrasonography were done for assessing the change in the thickness (depth) of the lesion. Visual analog scale score of pain and pruritis were also noted in all patients.

*Results:* We found that there is significant reduction in the thickness of recalcitrant keloids after applying 1 mCi of Y-90 skin patch in this single-arm trial ($p < 0.01$). The percent change from baseline on first, second, and third follow-up was 28, 32, and 48%, respectively, for 24 recalcitrant keloids. There is no significant decrease in the pruritis upon patch application over 6 months. The pain could not be commented upon because many patients had no pain during the baseline evaluation. Four lesions (very bulky lesions) had a recurrence over the follow-up period of 6 months. Side-effect profile: Most patients had initial skin ulcer and subsequently developed into hypopigmentation.

*Conclusion:* The decrease in thickness implies the effect of the Y-90 skin patch on recalcitrant keloids. There was an optimum change of thickness from baseline with 6 months follow-up. Few recurrences ($n = 4$) up to 6 months was observed during the follow-up. All the patients experienced subjective symptomatic relief in pruritis after the patch therapy. Therefore Y-90 skin patch may be considered as a cheap, noninvasive, effective treatment for recalcitrant keloids.
